# Near-Infrared Spectroscopy for Oedema Quantification: An Ex Vivo Porcine Skin Model

**DOI:** 10.3390/s25226971

**Published:** 2025-11-14

**Authors:** Mariana Castro-Montano, Meha Qassem, Panayiotis A. Kyriacou

**Affiliations:** Research Centre for Biomedical Engineering, City St George’s, University of London, London EC1V 0HB, UK; meha.qassem@citystgeorges.ac.uk (M.Q.); p.kyriacou@citystgeorges.ac.uk (P.A.K.)

**Keywords:** near-infrared spectroscopy, oedema monitoring, PLS

## Abstract

**Highlights:**

**What are the main findings?**

- NIRS proved to be a promising technique for oedema assessment, as it was sensitive to changes in water content within the tissue. This study establishes proof-of-concept for the use of NIRS in non-invasive oedema quantification.
- The ex vivo porcine skin model was shown to be a suitable and reproducible platform for inducing and studying oedema.

**What is the implication of the main findings?**

- These findings highlight the potential of NIRS for future in vivo applications. Ongoing work aims to validate this technique in a clinical study involving neonates with oedema, which could enable earlier diagnosis, improved treatment evaluation, and better patient management in conditions related to abnormal fluid accumulation.
- The porcine ex vivo model serves as a valuable intermediate step between controlled laboratory investigations and in vivo clinical validation.

**Abstract:**

Oedema is a common clinical finding in critically ill neonates and may reflect systemic illness such as congestive heart failure, hepatic cirrhosis, nephrotic syndrome, sepsis, and acute kidney injury. Oedema is characterised by tissue swelling due to water accumulation in the interstitial space. Currently, the gold standard in clinical practice is visual assessment, which is subjective and limited in accuracy. Alternative methods, such as ultrasound and bioimpedance, have been explored; however, they are unsuitable in neonates and do not provide direct water quantification. Near-infrared spectroscopy (NIRS) is a non-invasive optical method that could measure water content through light interaction between near-infrared light and OH particles within the tissue. This study validated NIRS for oedema assessment using an ex vivo porcine skin model, where controlled oedema was induced by phosphate-buffered saline (PBS) injection. Continuous spectroscopic data were collected via optical fibres positioned perpendicularly and parallel to the tissue. Regression models were developed and evaluated using the spectral data, with partial least squares (PLS) regression outperforming ridge regression (RR) and support vector regression (SVR). Notably, spectra acquired in the parallel configuration yielded superior results (R^2^ = 0.97, RMSE = 0.15). These findings support the potential of NIRS as a reliable, quantitative tool for neonatal oedema assessment.

## 1. Introduction

Oedema is characterised by skin swelling due to water accumulation in tissues. This condition is common in neonates in the intensive care unit [1], especially those with low weight or preterm birth [2,3]. Oedema is associated with underlying conditions such as heart failure, hepatic cirrhosis, nephrotic syndrome, sepsis, and acute kidney injury [4,5,6,7]. Furthermore, it can lead to clinical consequences, including deficient tissue perfusion and oxygenation, an increased risk of infections through the skin, and low levels of serum sodium and albumin [8,9,10,11].

Currently, clinical methods for oedema assessment in neonates rely on weight-based formulas, fluid input-output measurements [12,13]. However, critically ill neonates may be too unstable to weigh daily, and quantifying fluid outputs such as urine, stool, or vomit is often impractical [14]. Visual assessment and pitting test are also common practices; the test consists of applying pressure to the swollen area to assess whether a persistent indentation remains, indicating the presence of oedema [15,16,17]. However, both visual assessment and pitting test are subjective, unstandardized, highly reliant on clinician experience, and lack sensitivity for early detection [15,18,19].

Furthermore, non-invasive and bedside techniques for fluid balance distribution and, therefore, oedema have been explored. These methods include bioimpedance [20,21,22,23,24,25,26], ultrasound [27,28,29], near-infrared spectroscopy (NIRS) [30,31,32], magnetic resonance imaging (MRI) [33], dual-energy X-ray absorptiometry (DXA) [34,35], and air displacement plethysmography (ADP) [36]. While promising, bioimpedance and ultrasound are generally unsuitable for critically ill neonates and do not directly measure water content [37,38,39]. Particularly, bioimpedance analysis could offer a promising alternative, as it is low-cost, non-invasive, and fast [40]; nevertheless, its application in neonates is challenging due to the difficulty in electrode placement, and the need for the baby to remain motionless in a specific position for accurate data acquisition [37,38]. Ultrasound is limited by the absence of a standardised protocol; current approaches, such as measuring tissue velocity, B-lines, or tissue thickness, are indirect indicators and often yield variable results [27,39]. ADP, MRI, and DXA can provide comprehensive body composition metrics, such as fat and fat-free mass. However, this cannot specifically quantify water content and often requires patients to be moved to specialised equipment, posing logistical and safety challenges [41].

NIRS presents a promising, non-invasive alternative for oedema assessment, offering the advantages of being quick and easy to perform. A typical NIR spectrum of human skin is characterised by various bands associated with absorption of light by O–H, C–H, and N–H functional groups. Strong absorption bands of O–H, which can be directly linked to dermal water content [42], are observed within the NIR region at 970 nm, 1200 nm, 1450 nm, and 1900 nm [32,43,44]. This makes NIRS a potentially valuable tool for quantifying tissue water content and, consequently, oedema. However, further research is needed to fully validate its clinical utility and establish its effectiveness for routine oedema detection and monitoring.

Due to the difficulties in collecting data from patients with oedema and the lack of control inherent in in vivo or clinical studies, an ex vivo model was developed using porcine skin to simulate oedema. Oedema was induced by delivering an intradermal injection of phosphate-buffered saline (PBS) solution, allowing for controlled and repeatable fluid administration. The main aim of this study is to investigate the relationship between spectroscopic measurements in the near-infrared region and the volume of solution injected into porcine skin at varying levels, with the objective of exploring the potential for oedema quantification. By analysing how spectral features change in response to controlled fluid injection, the study seeks to establish a reliable, non-invasive method for detecting and quantifying oedema based on optical measurements.

## 2. Materials and Methods

Porcine skin was selected as an ex vivo model due to its close similarity to human skin in terms of optical properties such as scattering and absorption. This tissue type has been used in previous optical and biomedical studies as a reliable substitute for human skin [42,45].

Porcine skin samples were prepared by cutting them to appropriate sizes and thoroughly cleaning them with multiple washes of ethanol to eliminate surface contaminants. The skin was then tap-dried using paper towels. Phosphate-buffered saline (PBS) solution was prepared by diluting one tablet of PBS (Fisher Scientific, Leicestershire, UK) in 100 mL of distilled water. PBS was selected over pure water to maintain physiological osmolarity and prevent osmotic imbalances within the tissue. To induce oedema, PBS was injected into the tissue.

A hypodermic needle (21 G 0.8 × 16 mm, BD Discardit™ II, Berkshire, UK) was inserted at an angle of approximately 30° to the skin surface to a depth of 9 mm. Using a fluidic pump (Legato^®^ 180, KDScientific, Holliston, MA, USA) connected to a 10 mL syringe with a luer lock (Fisher Scientific, Leicestershire, UK), PBS was delivered at a controlled flow rate of 300 µL/min until a total volume of 3 mL was administered. To validate that oedema was being formed in a controlled and localised manner, a solution of 1:100 methylene blue dye/H2O (BDH Limited, Poole, UK) was used in a separate trial. A total of 2 mL of methylene blue solution was injected into the tissue in 0.5 mL increments. This ex vivo model protocol was adapted from the method described in [46].

**Figure 1:**
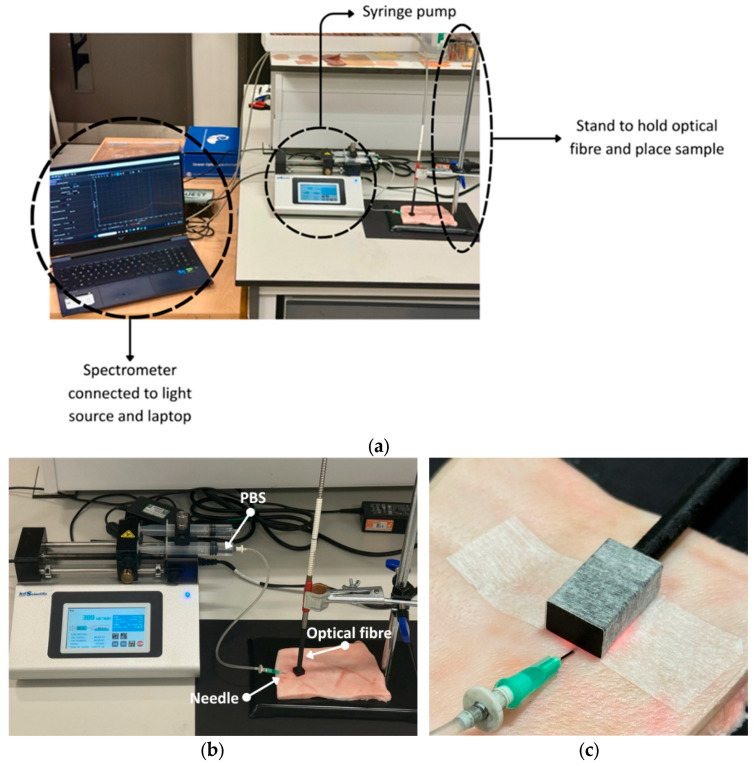
Experiment setup. (**a**) Connection of NIRQUEST512 near-infrared spectrometer, syringe pump, and laptop. (**b**) Placement of the optical fibre in the perpendicular position and the needle on the sample with PBS injection. (**c**) Placement of the optical fibre in the parallel position.

NIRQuest512 near-infrared spectrometer (Ocean Optics, Orlando, FL, USA) was utilised to acquire spectral measurements, covering the range of 900–1700 nm. The device was connected to a halogen light source (HL-2000, Ocean Optics, Orlando, FL, USA) and a laptop for data acquisition, with OceanView Software (version 2.0.19) employed for spectral data recording and visualisation. A bifurcated optical fibre (Ocean Optics, Orlando, FL, USA) was used. The optical fibre was positioned on top of the skin, near the site of needle insertion. Spectroscopic data were recorded continuously throughout the injection process (integration time = 100 ms). Baseline measurements were captured prior to the initiation of fluid delivery (0 mL) to provide reference data. Figure 1 illustrates the experimental setup.

Two different attachments were designed to position the optical fibre for spectral data acquisition. The first attachment allows the optical fibre to be placed perpendicular to the skin (Figure 1b), following the traditional method used in optical fibre spectroscopy. This attachment ensures a safe and consistent contact with the skin without applying pressure to the tissue, while maintaining a fixed distance of 1 mm between the fibre tip and the tissue surface. The second attachment positions the optical fibre parallel to the skin (Figure 1c). It includes an internal silver mirror (Thorlabs, Ely, UK) angled at 45°, which redirects the light to strike the skin at a 90° angle. The reflected light then follows the same optical path back. The objective of using both attachments is to compare their performance in terms of usability and data quality, and to assess whether oedema can be reliably detected and quantified with each configuration. In total, three porcine samples were analysed. For two samples, spectral data were recorded with the optical fibre positioned perpendicular, while for one sample, the parallel configuration was used.

Two independent analyses were performed. The first involved the spectral feature extraction and quantitative comparison between the perpendicular and the parallel configurations, while the second focused on the development and evaluation of regression models.

For quantitative comparison between the perpendicular and parallel configurations, the signal-to-noise ratio (SNR) was calculated at the water absorption peaks (970, 1200, and 1450 nm), using, as the noise, the spectral regions free of water absorption and dominated by instrument noise (900–950 nm and 1650–1700 nm). In addition, the correlation coefficient, best-fit amplitude scaling factor, and relative residual norm were computed to evaluate overall spectral similarity between the two orientations.

Regarding data processing and feature extraction, spectral data were smoothed using a Savitzky–Golay (SG) filter with a 2nd-order polynomial and a window size of 11. A 2nd-order polynomial still retains the basic curvature of the band’s structure and was previously applied to similar data in [42], and a window size of 11 is large enough to suppress high-frequency noise, yet small enough not to broaden or distort narrow spectral features relevant to our analysis. From the OH-related bands at 1200 nm and 1450 nm, spectral features such as peak amplitude, width, and area under the curve were extracted. These features were extracted from mean spectra, which were calculated over successive fluid volume intervals (0–0.5 mL, 0.5–1.0 mL, …, up to 2.5–3.0 mL), yielding a total of seven mean spectra.

Furthermore, for data analysis, regression models including Partial Least Squares (PLS), Support Vector Regression (SVR), Ridge Regression (RR), and Ridge Regression combined with Principal Component Analysis (PCA) were evaluated using the spectral data. PLS is considered the gold standard in chemometrics analysis, while SVR and RR are well-suited for handling multicollinear data. To ensure reliable performance, model parameters were optimised, with the coefficient of determination (R^2^) used as the primary selection criterion. Table 1 lists the parameter values explored during the optimisation process for each model. After optimisation, model performance was assessed and compared using multiple error metrics, including mean squared error (MSE), root mean squared error (RMSE), and mean absolute error (MAE).

RMSE emphasises larger errors due to the squaring of residuals, making it particularly useful when large deviations are more critical, and its interpretation is easier since it provides an error measure in the same unit as the target variable [47]. For this reason, RMSE was chosen as the primary metric for performance comparison. MAE provides a measure of the average magnitude of errors regardless of direction and is less sensitive to outliers, and it is one of the most widely used performance metrics for evaluation of a regression model [47], while MSE was primarily used for completeness and consistency with common statistical reporting, but it is less interpretable in the original units of the target variable.

Prior to modelling, the spectra were smoothed with an SG filter. Because of the large number of wavelengths, only the range of 1000–1600 nm was used for analysis, as the spectrum edges were noisy, and this range captures the two water-related peaks of interest.

To evaluate model performance and generalizability, the dataset was partitioned into multiple training and testing sets using four different data split strategies, as summarised in Table 2.

The dataset was divided into seven groups based on the volume of fluid injected: group 0 included baseline data with 0 mL injected; group 1 contained data from 0 mL to 0.5 mL; group 2 from 0.5 mL to 1.0 mL; group 3 from 1.0 mL to 1.5 mL; group 4 from 1.5 mL to 2.0 mL; group 5 from 2.0 mL to 2.5 mL; and group 6 from 2.5 mL to 3.0 mL. Four different data splits were created for training and testing using the groups created. In the first split, groups 0, 1, 2, 4, and 6 were used for training, while groups 3 and 5 were reserved for testing. The second split used groups 0, 1, 3, 5, 6 for training and groups 2 and 4 for testing. The third split consisted of training on groups 0, 1, 2, 3, and 5, and testing on groups 4 and 6. Finally, the fourth split excluded the extreme values, using only groups 1, 2, 3, 4, and 5 for training and reserving groups 0 and 6 for testing.

Specifically, the dataset was divided according to incremental injection fluid volumes to evaluate the model’s ability to generalise across different physiological states. The four data splits were designed to test both interpolation and extrapolation performance by systematically including or excluding certain injection volume ranges. This approach ensures that the model’s predictive capacity is not limited to interpolation within known data ranges, but it is also challenged to predict outside them.

Cross-validation (CV) was used in model training, implementing venetian blinds with 5 splits and a thickness of 50. A vector of fluid injected was added to the data. It was calculated using the time vector and the injection rate.

**Figure 2:**
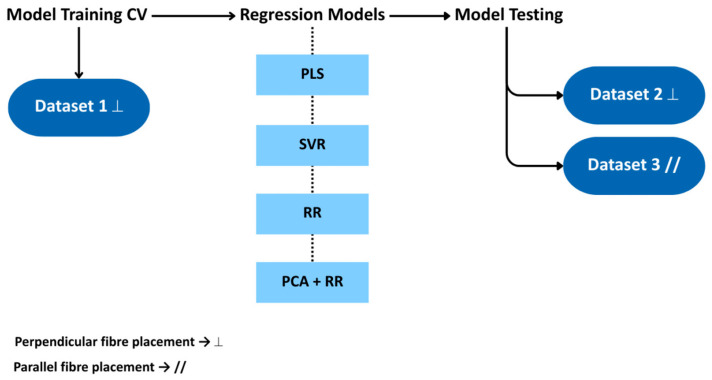
Regression models scheme of data training and testing using different datasets. PLS = partial least squares; SVR = support vector regression; RR = ridge regression; PCA = principal component analysis; CV = cross-validation.

In addition, regression models were trained on the full dataset and validated using two independent test datasets, as shown in Figure 2. The training dataset consisted of spectral measurements acquired with the optical fibre positioned perpendicular to the sample. For testing, one dataset was collected using the same perpendicular configuration, while the other was acquired with the fibre oriented parallel to the sample.

## 3. Results

### 3.1. Spectral Features

**Figure 3:**
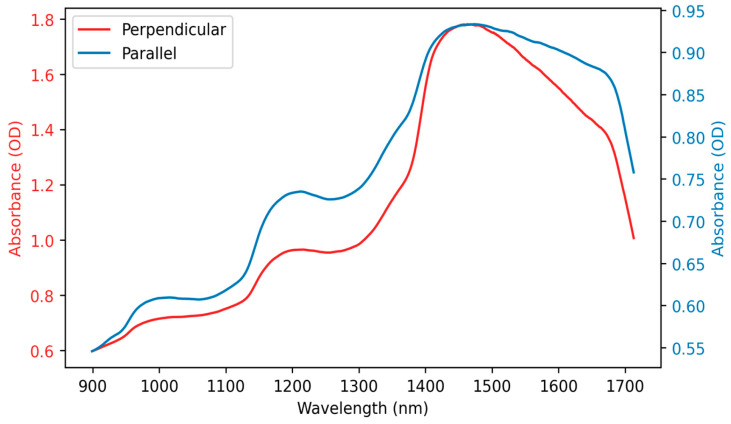
Spectrum acquired, placing the optical fibre in the perpendicular position (red) compared to the spectrum acquired in the parallel position (blue). OD = optical density.

Data was collected from the same sample and at the same location using the two attachments designed to position the optical fibre either perpendicular or parallel to the tissue surface. Figure 3 displays the resulting spectra. Both spectra exhibit the characteristics of the water absorption bands at 970, 1200, and 1450 nm, and while the overall shapes of the two spectra are similar, their total signal amplitudes differ. SNRs were measured at these water peaks; for a perpendicular spectrum, the SNRs are 1.99, 2.77, and 5.09 at 970, 1200, and 1450 nm, respectively, whereas for the parallel spectrum, the values are higher by 1.99, 2.77, and 5.09.

Furthermore, the spectra exhibit a correlation coefficient of 0.98, a best-fit scaling factor of 0.30, and a relative residual norm of 28.25%. These metrics confirm that the overall spectral dynamics and relative peak structure are preserved between the two orientations; however, there are small variations likely caused by light losses in the parallel configuration, resulting from the longer optical path and reflection from the mirror.

**Figure 4:**
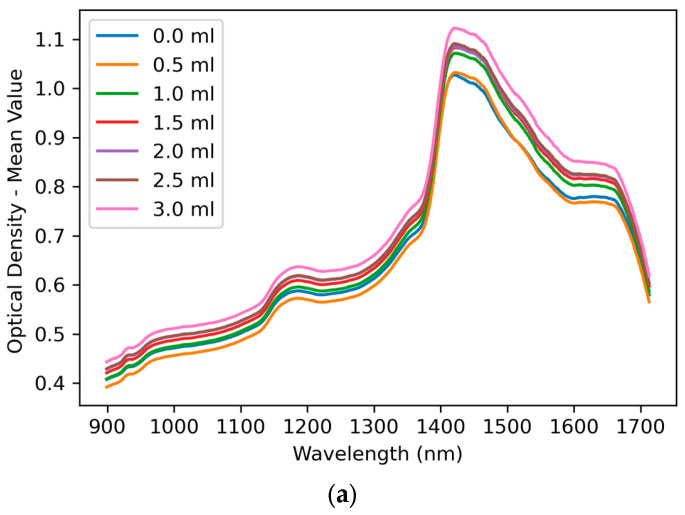

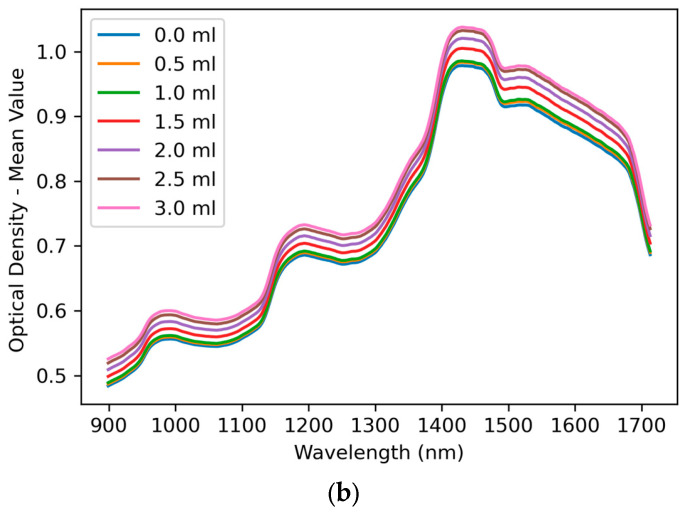
Mean optical density at different fluid injection volumes. The fluid increased from 0 mL to 3 mL in steps of 0.5 mL. Spectra acquired with the fibre oriented (**a**) perpendicular and (**b**) parallel to the tissue in the wavelength range of 900–1700 nm.

The mean spectra were calculated and plotted for each fluid volume increment, ranging from 0 mL to 3 mL in steps of 0.5 mL, as illustrated in Figure 4. The resulting plots showed a clear trend of increasing spectral amplitude with higher volumes of injected fluid, with both perpendicular and parallel data acquisition. This effect was particularly noticeable at wavelengths corresponding to known water absorption peaks, indicating a progressive increase in water content within the tissue as fluid volume increased. These spectral changes further support the sensitivity of the measurement setup to detect oedema-related variations.

**Figure 5:**
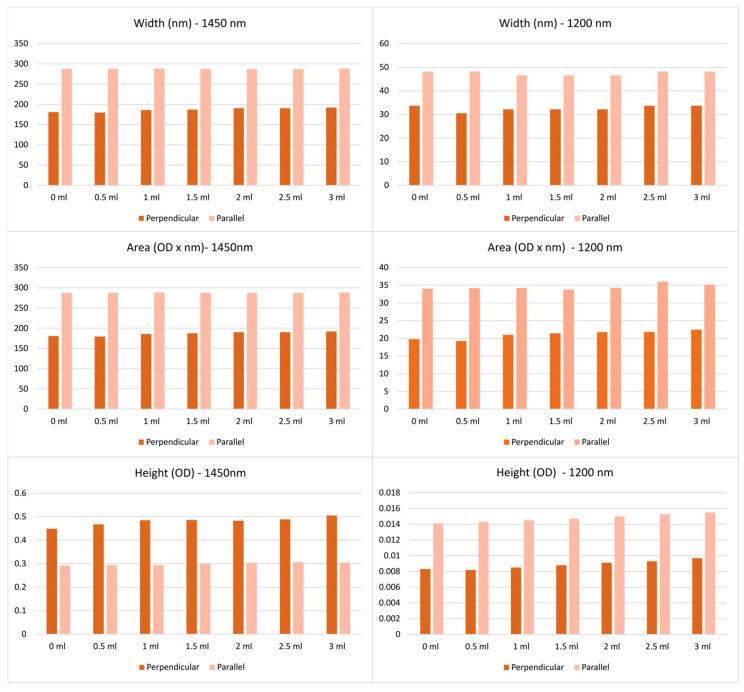
Height, width, and area under the curve for perpendicular and parallel measurements across different fluid volumes (0–3 mL, in 0.5 mL increments) at 1200 nm and 1450 nm. Top row: width measurements (nm); middle row: area (OD × nm); bottom row: height (OD). The left column corresponds to 1450 nm and the right column to 1200 nm. OD = optical density.

Furthermore, spectral features, such as peak amplitude, width, and area under the curve, were extracted at 1200 nm and 1450 nm peaks from data acquired using both the perpendicular and parallel approaches. As shown in Figure 5, the perpendicular configuration yielded smaller widths and areas for both peaks compared to the parallel configuration. For peak height, the parallel approach produced higher values at 1200 nm, whereas the perpendicular approach showed higher values at 1450 nm. Despite these differences, all three features exhibited the same overall trend in both acquisition methods; their values increased with increasing fluid volume.

### 3.2. Regression Models

Table 3 summarises the performance of the regression models during training and testing across different splits, along with the parameters found during optimisation, using spectral data acquired with the perpendicular fibre placement approach. Overall, PLS achieved the most consistent and reliable performance, with the lowest error metrics (RMSE between 0.116 and 0.283) and the greatest stability across splits. SVR also performed strongly during training, but its testing results were more variable (R^2^ between 0.7891 and 0.9512), indicating greater sensitivity to data splits and less robust generalisation compared to PLS. RR, in contrast, performed considerably worse, with lower R^2^ values during cross-validation and very high testing errors (RMSE between 0.332 and 0.7601). However, applying PCA before RR improved its performance (RMSE between 0.1384 and 0.265), bringing it closer to that of PLS and SVR, although variability across splits was still observed.

Similarly, Table 4 presents the performance of the regression models using data acquired with the parallel fibre placement approach. In this case, PLS again outperformed the other models, delivering consistent and strong results across splits (RMSE between 0.0772 and 0.1272). SVR achieved similarly high R^2^ values during cross-validation (R^2^ up to 0.997) and produced strong testing results, although with slightly less stability than PLS (RMSE between 0.089 and 0.169). RR performed reasonably in some splits but remained less reliable than PLS or SVR, showing greater variability (R^2^ between 0.75 and 0.96) and larger error values (RMSE up to 0.2592). Incorporating PCA further improved RR, reducing errors and enhancing overall performance (RMSE between 0.0924 and 0.1761).

Both PLS and SVR performed better with parallel fibre placement than with perpendicular placement, showing higher stability and lower errors. RR also improved in the parallel case but remained the weakest method, while PCA + RR continued to deliver stronger performance than RR alone.

Additionally, the regression models were trained on a full dataset and validated using two independent test datasets. As presented in Table 5, PLS demonstrated strong cross-validation performance and consistent reliability across both configurations, achieving better results with the parallel setup (R^2^ = 0.9742, RMSE = 0.1499) than with the perpendicular setup (R^2^ = 0.9273, RMSE = 0.252). SVR also performed well, though it proved slightly more sensitive to acquisition orientation, since performance was weaker with perpendicular data (R^2^ = 0.9156, RMSE = 0.2714) compared to parallel data (R^2^ = 0.9565, RMSE = 0.1946). RR showed comparable cross-validation results to SVR and generalised reasonably well, again with superior performance for parallel data (R^2^ = 0.9645, RMSE = 0.1758) compared to perpendicular (R^2^ = 0.9192, RMSE = 0.2657). In contrast, applying PCA prior to RR led to unstable behaviour, performing adequately with perpendicular data but failing in the parallel case (negative R^2^ = −2.818, RMSE = 1.82).

Taken together, these results confirm that PLS achieved the best and most consistent performance, while all models, except PCA + RR, performed better with data acquired using the parallel configuration.

Moreover, PLS outperformed both SVR and RR across Table 3, Table 4 and Table 5. Incorporating PCA into RR improved its performance; however, PLS remained the most suitable approach for this type of data, as expected given its proven effectiveness in chemometric applications [48]. While SVR usually performs equally well as PLS, one of its key advantages is its robust performance with limited calibration data, a feature particularly valuable in real-world applications [49].

## 4. Discussion

This study demonstrates that the ex vivo porcine skin model offers a suitable and reproducible platform for inducing oedema. Controlled PBS injection enabled physiologically relevant fluid accumulation while ensuring experimental consistency. As such, this model provides a valuable intermediate step between laboratory studies and in vivo validation.

Similarly, NIRS proved to be a promising technique for oedema assessment, as it was sensitive to changes in water content within the tissue. The observed changes in spectral features, such as peak amplitude, width, and area under the curve, particularly in regions associated with water absorption around 1200 nm and 1450 nm, confirmed the capacity of NIRS to detect and quantify dermal fluid accumulation. These findings are consistent with those of Budylin et al. [30], who observed increased amplitude of the 1200 nm band with oedema progression. The reproducibility of this trend across our measurements strengthens the evidence that these spectral markers can serve as reliable indicators of tissue oedema.

Both perpendicular and parallel fibre configurations enabled the acquisition of high-quality spectral data and captured oedema-related spectral changes with comparable trends. In both setups, the absorbance bands around 1200 nm and 1450 nm increased as fluid was introduced. The parallel configuration, although showing a slight reduction in spectral amplitude, preserved the overall shape and dynamics of the spectra, indicating that it retained the essential information required for modelling. The parallel configuration may offer practical advantages in real-world scenarios, where probe positioning could be constrained by anatomical or clinical factors.

In terms of predictive modelling, PLS regression consistently outperformed other methods. This result was expected, as PLS is considered the gold standard in NIRS analysis [48,50], due to its robustness in handling collinear spectral data and its capacity to extract valuable chemical information from NIR spectral data [51], even when used in small sample datasets [52].

PLS regression model showed strong performance not only during interpolation within the training data range but also during extrapolation to fluid volumes outside the immediate training set. Importantly, PLS generalised effectively to an independent dataset collected from a separate tissue sample, yielding high R^2^ values and low error metrics. This performance underscores both the stability of the extracted spectral data and the robustness of the model. By contrast, while SVR and RR also performed well in certain scenarios, their stability was more sensitive to acquisition conditions and data splits. The failure of PCA combined with RR in the parallel dataset underscores the importance of selecting appropriate modelling parameters, suggesting that the optimal number of principal components may differ between data acquired with the perpendicular and parallel configurations. Because the model was trained on perpendicular data, it performs better when tested on data from the same perpendicular configuration, compared to data from the parallel configuration.

A key observation is that regression models trained with parallel data consistently yielded better results than those trained with perpendicular data. Beyond the quantitative improvements, the parallel configuration also offered practical advantages such as easier fibre placement, reduced sensitivity to positioning errors, and greater potential for stable data acquisition. These characteristics make it particularly suitable for clinical applications, where probe positioning is often influenced by patient movement, anatomy, or external constraints. Importantly, the comparable spectral trends between perpendicular and parallel configurations suggest that no essential spectral information is lost with the parallel setup, making it the more practical choice for translation into in vivo and clinical studies.

Previous work by Stamatas et al. [32] also explored oedema detection using NIRS. Compared to their approach, our method enables the detection of subtle changes in tissue water content, even when fluid accumulation is present, but swelling is not yet visible. Stamatas et al. [32] relied on changes in tissue surface and estimated oedema volume from the swollen area, without analysing light absorption at specific wavelengths. Their system operated in the 400–970 nm range, where water absorption is only significant near 970 nm. In contrast, we used a spectrometer that covers 900–1700 nm, including three water-related absorption bands (970, 1200, and 1450 nm), which enables a more sensitive and quantitative assessment of water content through multivariate analysis.

To date, studies on fluid accumulation have not directly quantified tissue water content, and clinically, there are no standardised reference values of tissue water content to determine whether a patient is oedematous or to assess the severity of oedema. Although some reports suggest that a fluid accumulation exceeding 10% may be considered pathological [13]. Our study represents an initial step toward quantifying oedema, and future in vivo studies using this technique could help establish a baseline threshold for oedema classification.

Overall, this study establishes proof-of-concept for the use of NIRS in non-invasive oedema quantification and validates porcine skin as a reliable surrogate for preliminary model development. The strong and consistent performance of PLS regression, combined with the robustness of parallel probe placement, highlights the potential of this approach for future in vivo applications.

Our future work consists of validating this technique in vivo in a clinical study on neonates with oedema. The current ex vivo experiments were designed to mimic key aspects of the clinical scenario, such as optical fibre placement, and allowed us to confirm that the device functions reliably. The ex vivo findings provide essential guidance for in vivo studies, guiding the selection of pre-processing parameters and model architectures. This foundation ensured that the transition to clinical applications is based on validated device performance and a clear understanding of spectral signatures. The in vivo study results could enable real-time monitoring of oedema progression, support early diagnosis, treatment evaluation, and improve patient management in conditions associated with abnormal fluid accumulation.

This study has some limitations, including the limited sample size, the lack of comparison with other techniques such as bioimpedance and ultrasound, and the exclusion of physiological factors (e.g., skin temperature) that may influence or confound oedema detection. Future in vivo studies addressing these aspects will provide valuable insights into the potential advantages of NIRS for oedema detection.

## Figures and Tables

**Table 1:** Parameters for grid search optimisation. PLS = partial least squares; SVR = support vector regression; RR = ridge regression; LV = latent variables; C = regularisation parameter.

Model	Parameters for Optimisation
PLS	‘LV’: range from 1 to 20.
SVR	‘kernel’: [‘linear’, ‘poly’, ‘rbf’, ‘sigmoid’], ‘gamma’: [‘scale’, ‘auto’, 0.01, 0.1, 1], ‘C’: [0.1, 1]
RR	‘alpha’: [1, 10, 100], ‘solver’: [‘svd’, ‘cholesky’, ‘lsqr’, ‘sparse_cg’, ‘sag’, ‘saga’]

**Table 2:** Injection volume groups and data splitting for model training (blue cells) and testing (yellow cells).

Groups	Range	Split 1	Split 2	Split 3	Split 4
0	0 ml				
1	0 mL to 0.5 mL				
2	0.5 mL to 1 mL				
3	1 mL to 1.5 mL				
4	1.5 mL to 2 mL				
5	2 mL to 2.5 mL				
6	2.5 mL to 3 mL				

**Table 3:** PLS, SVR, and RR performance during CV and testing using data from the perpendicular approach. PLS = partial least squares; SVR = support vector regression; RR = ridge regression; PCA = principal component analysis; LV = latent variable; CV = cross-validation; R^2^ = coefficient of determination; MSE = mean squared error; RMSE = root mean squared error; MAE = mean absolute error; sd = standard deviation; C = regularisation parameter.

			CV Results (Mean ± sd)		Testing Results			
Model	Splits	Optimal Parameters	R^2^	MSE	R^2^	MSE	RMSE	MAE
PLS	1	5 LV	0.9851 ± 0.0015	0.0164 ± 0.0015	0.9503	0.0135	0.1160	0.0986
	2	5 LV	0.9857 ± 0.0027	0.0166 ± 0.0030	0.8929	0.0290	0.1703	0.1341
	3	5 LV	0.9858 ± 0.0024	0.0091 ± 0.0019	0.8937	0.0514	0.2268	0.1971
	4	6 LV	0.9861 ± 0.0024	0.0072 ± 0.0013	0.9551	0.0803	0.2834	0.2601
SVR	1	C: 1, gamma: 0.1, kernel: ‘poly’	0.9907 ± 0.0016	0.0102 ± 0.0019	0.9334	0.0180	0.1343	0.1103
	2	C: 1, gamma: 0.1, kernel: ‘poly’	0.9914 ± 0.0022	0.0101 ± 0.0027	0.9160	0.0227	0.1508	0.1321
	3	C: 1, gamma: 0.1, kernel: ‘poly’	0.9869 ± 0.0027	0.0084 ± 0.0018	0.7891	0.0615	0.2480	0.1933
	4	C: 1, gamma: 0.1, kernel: ‘poly’	0.9819 ± 0.0050	0.0095 ± 0.0029	0.9512	0.0871	0.2952	0.2373
RR	1	alpha’: 1, ‘solver’: ‘cholesky’	0.8564 ± 0.0061	0.1587 ± 0.0098	0.2604	0.2003	0.4475	0.3805
	2	alpha’: 1, ‘solver’: ‘sag’	0.8292 ± 0.0223	0.1995 ± 0.0297	0.5928	0.1103	0.3321	0.2709
	3	alpha’: 1, ‘solver’: ‘cholesky’	0.7082 ± 0.0278	0.1858 ± 0.0171	0.3748	0.1824	0.4271	0.3422
	4	alpha’: 1, ‘solver’: ‘sag’	0.7795 ± 0.0339	0.1150 ± 0.0216	0.6767	0.5778	0.7601	0.6792
PCA + RR	1	alpha’: 1, ‘solver’: ‘svd’	0.9891 ± 0.0008	0.0120 ± 0.0010	0.9292	0.0192	0.1384	0.1195
	2	alpha’: 1, ‘solver’: ‘svd’	0.9913 ± 0.0008	0.0101 ± 0.0010	0.9026	0.0264	0.1624	0.1420
	3	alpha’: 1, ‘solver’: ‘cholesky’	0.9828 ± 0.0027	0.0110 ± 0.0021	0.8245	0.0512	0.2263	0.1925
	4	alpha’: 1, ‘solver’: ‘svd’	0.9824 ± 0.0032	0.0092 ± 0.0019	0.9608	0.0701	0.2648	0.2405

**Table 4:** PLS, SVR, and RR performance during CV and testing using data from the parallel approach. PLS = partial least squares; SVR = support vector regression; RR = ridge regression; PCA = principal component analysis; LV = latent variable; CV = cross-validation; R^2^ = coefficient of determination; MSE = mean squared error; RMSE = root mean squared error; MAE = mean absolute error; sd = standard deviation; C = regularisation parameter.

			CV Results (Mean ± sd)		Testing Results			
Model	Splits	Optimal Parameters	R^2^	MSE	R^2^	MSE	RMSE	MAE
PLS	1	5 LV	0.9971 ± 0.0005	0.0030 ± 0.0005	0.9780	0.0060	0.0772	0.0648
	2	5 LV	0.9974 ± 0.0003	0.0029 ± 0.0003	0.9693	0.0083	0.0912	0.0739
	3	5 LV	0.9964 ± 0.0004	0.0023 ± 0.0002	0.9402	0.0162	0.1272	0.0965
	4	5 LV	0.9960 ± 0.0002	0.0021 ± 0.0002	0.9913	0.0157	0.1253	0.1013
SVR	1	C: 1, gamma: ‘scale’, kernel: ‘poly’	0.9963 ± 0.0005	0.0038 ± 0.0005	0.9707	0.0079	0.0891	0.0733
	2	C: 1, gamma: ‘scale’, kernel: ‘poly’	0.9966 ± 0.0005	0.0038 ± 0.0005	0.9665	0.0091	0.0953	0.0772
	3	C: 1, gamma: ‘scale’, kernel: ‘poly’	0.9949 ± 0.0008	0.0032 ± 0.0004	0.9162	0.0227	0.1505	0.1150
	4	C: 1, gamma: ‘scale’, kernel: ‘poly’	0.9940 ± 0.0002	0.0031 ± 0.0001	0.9841	0.0287	0.1693	0.1369
RR	1	alpha’: 1, ‘solver’: ‘saga’	0.9741 ± 0.0017	0.0267 ± 0.0012	0.9202	0.0216	0.1471	0.1279
	2	alpha’: 1, ‘solver’: ‘lsqr’	0.9865 ± 0.0022	0.0151 ± 0.0021	0.7518	0.0672	0.2592	0.1951
	3	alpha’: 1, ‘solver’: ‘saga’	0.9540 ± 0.0032	0.0293 ± 0.0006	0.9041	0.0259	0.1610	0.1140
	4	alpha’: 1, ‘solver’: ‘sag’	0.9591 ± 0.0026	0.0213 ± 0.0016	0.9631	0.0664	0.2577	0.2449
PCA + RR	1	alpha’: 1, ‘solver’: ‘cholesky’	0.9951 ± 0.0011	0.0050 ± 0.0012	0.9678	0.0087	0.0934	0.0784
	2	alpha’: 1, ‘solver’: ‘svd’	0.9953 ± 0.0010	0.0053 ± 0.0011	0.9685	0.0085	0.0924	0.0751
	3	alpha’: 1, ‘solver’: ‘svd’	0.9937 ± 0.0012	0.0040 ± 0.0007	0.8940	0.0287	0.1693	0.1340
	4	alpha’: 1, ‘solver’: ‘svd’	0.9930 ± 0.0004	0.0037 ± 0.0001	0.9828	0.0310	0.1761	0.1433

**Table 5:** PLS, SVR, and RR performance during CV using data from the perpendicular approach and testing using data from the perpendicular and parallel approaches. PLS = partial least squares; SVR = support vector regression; RR = ridge regression; PCA = principal component analysis; LV = latent variable; CV = cross-validation; R^2^ = coefficient of determination; MSE = mean squared error; RMSE = root mean squared error; MAE = mean absolute error; sd = standard deviation; C = regularisation parameter.

		CV Results (Mean ± sd)			Testing Results			
Model	Optimal Parameters	R^2^	MSE	Dataset Used for Testing	R^2^	MSE	RMSE	MAE
PLS	5 LV	0.9800 ± 0.0044	0.0182 ± 0.0041	Perpendicular	0.9273	0.0635	0.2520	0.2084
				Parallel	0.9742	0.0225	0.1499	0.1217
SVR	C: 1, gamma: 0.1, kernel: ‘linear’	0.9915 ± 0.0010	0.0077 ± 0.0011	Perpendicular	0.9156	0.0737	0.2714	0.2194
				Parallel	0.9565	0.0379	0.1946	0.1590
RR	alpha’: 1, ‘solver’: ‘svd’	0.9904 ± 0.0013	0.0087 ± 0.0014	Perpendicular	0.9192	0.0706	0.2657	0.2156
				Parallel	0.9645	0.0309	0.1758	0.1437
RR + PCA	alpha’: 1, ‘solver’: ‘svd’	0.9871 ± 0.0020	0.0118 ± 0.0021	Perpendicular	0.8892	0.0967	0.3110	0.2170
				Parallel	−2.8180	3.3211	1.8224	1.6103

## Data Availability

Data available in a publicly accessible repository https://github.com/mariana-castro-montano/ExViVoOedema.
